# A randomized controlled trial on a multicomponent intervention for overweight school-aged children – Copenhagen, Denmark

**DOI:** 10.1186/1471-2431-14-273

**Published:** 2014-10-21

**Authors:** Nina Majlund Harder-Lauridsen, Nina Marie Birk, Mathias Ried-Larsen, Anders Juul, Lars Bo Andersen, Bente Klarlund Pedersen, Rikke Krogh-Madsen

**Affiliations:** Department of Infectious Diseases The Centre of Inflammation and Metabolism and the Centre for Physical Activity Research, Rigshospitalet, the Faculty of Health and Medical Sciences, University of Copenhagen, Copenhagen, Denmark; Department of Growth and Reproduction, Rigshospitalet, the Faculty of Health and Medical Sciences, University of Copenhagen, Copenhagen, Denmark; Research Unit for Exercise Epidemiology, Institute of Sport Science and Clinical Biomechanics, Centre of Research in Childhood Health, University of Southern Denmark, Odense, Denmark

**Keywords:** Overweight children, Obesity, Randomized controlled trial, Multicomponent intervention, Physical activity, Diet counseling, Body mass index, Blood glucose, Insulin, Insulin resistance

## Abstract

**Background:**

Obesity amongst children is a growing problem worldwide. In contrast to adults, little is known on the effects of controlled weight loss on components of the metabolic syndrome in children. The primary aim of the study was to evaluate the effects of a 20-week exercise and diet guidance intervention on body mass index (BMI) in a group of overweight children. Our hypothesis was an observed reduction in BMI and secondarily in body fat content, insulin insensitivity, and other components of the metabolic syndrome in the intervention group.

**Methods:**

School children from Copenhagen were randomly allocated to an intervention group (n = 19) or a control group (n = 19). Anthropometric assessment, whole body dual-energy X-ray absorptiometry scan, two hours oral glucose tolerance test, steps measured by pedometer, and fitness tests were measured at baseline and at 20 weeks.

**Results:**

Thirty-seven children (30 girls) participated at baseline, aged 8.7 ± 0.9 years with a BMI of 21.8 ± 3.7 kg/m^2^ (mean ± SD), and 36 children completed the study. The intervention group decreased their BMI (the intervention effect is the difference in change between the groups adjusted for the respective baseline values (DELTA) = -2.0 kg/m^2^, 95% CI: -2.5; -1.5, P <0.001), total body mass (DELTA = -4.0 kg, 95% CI: -4.9; -3.0, P <0.001), and fat mass (DELTA = -3.3 kg, 95% CI: -4.2; -2.7, P <0.001) compared to the control group after the intervention. The intervention group displayed decreased waist, hip and waist-to-height ratio (WHtR) (all three variables; P <0.001), area under curve for plasma insulin (P <0.05), and increased mean and minimum steps/day (P <0.05 and P <0.01, respectively).

**Conclusions:**

The multicomponent intervention had significant favorable effects on BMI, weight, WHtR, mean and minimum steps/day, and fat mass. In addition, similar beneficial metabolic effects were found in the children as shown in adults, e.g. increase in peripheral insulin sensitivity.

**Trial registration:**

Clinicaltrials.gov Identifier number NCT01660789.

## Background

Overweight and obesity in children is a growing problem, and 170 million children below the age of 18 years globally are estimated to be overweight [1]. In adults, the combination of obesity and inactivity yields higher risks of a range of diseases, e.g. type 2 diabetes, certain types of cancer and cardiovascular diseases [2–4]. Physical inactivity and low physical fitness are associated with high abdominal fat, obesity, insulin resistance, and elevated blood lipid levels in children [5, 6] which contribute to the increasing prevalence of the metabolic syndrome [7–10]. There is an association between overweight in children and some of the risk factors of the metabolic syndrome [11], and a positive effect on these risk factors by physical activity [8]. In addition, energy intake has increased rapidly since the 1950s [12], and socio-economic status of the parents, parental overweight, and higher birth weight are all also associated with overweight children [13]. Overweight and obese children are furthermore at risk of a poor quality of life and early stigmatization with lower self-esteem, greater shame and perceived teasing compared with their non-overweight peers [14]. Hence, childhood obesity and inactivity are considered among the most serious threats to public health in the 21st century [2, 14]. Alarmingly, over 60% of the children who are overweight before puberty will also be overweight in early adulthood [15, 16]. Thus obesity needs to be addressed from an early age.

The few well-designed randomized controlled trials (RCT) on the reversal of overweight in childhood have proven most effective when targeting multiple risk behaviors (with focus on physical activity, diet and behavior change) [17, 18]. However, most have been conducted in the clinic and are labor intensive. Additional well-designed community-based studies are required with a strong family-based component included [17]. The multifactorial interventions must be cost-effective, feasible, and engaging for both children and their parents and furthermore, to reach long-term results, the intervention should include ongoing support from society as a whole, as well as family, school and government agencies [19].

The main purpose of the study was to evaluate a multicomponent intervention with a focus on diet and physical activity on a group of overweight children. The intervention was chosen based on the following criteria: 1) low-cost, 2) feasibility (with only two training sessions per week in a non-clinical setting), and 3) accessibility (e.g. training in the neighborhood and free access to fitness center) for both children and their families. Our hypothesis was to find a decrease in BMI supported by a decrease in fat mass and an increase in muscle mass (DXA-scans) in the intervention group compared to the control group. Furthermore, we expected to find increased fitness and strength, improvement in glucose and lipid metabolism, and profitable changes in plasma levels of adipokines.

## Methods

### Ethics statement

The parents and children were given both oral and written information about the study and the parents signed a consent form. The study was carried out in accordance with the Helsinki declaration and approved in advance (1 December 2011) by the Danish Ethics Committees of the Capital Region (record number H-2-2011-132).

This study was registered at the Danish Data Protection Agency (record number 2008-54-0474).

In addition, the study was registered at Clinicaltrials.gov (Identifier number NCT01660789).

The authors confirm that all ongoing and related trials for this intervention are registered.

### Participants and procedures

Postal letters were sent to parents in Høje-Taastrup Municipality near Copenhagen with children aged 7–10 years in regard to information from the ‘SundSkoleNettet’/‘The Healthy Schools Network’ (SSN). SSN is a national Danish network collecting yearly data on Danish public school children’s health status (measurements include weight, height, waist, jump height, aerobic fitness test, and steps registered with pedometer), with the aim to monitor the health of children. Parents with overweight children (n = 867) were invited to participate in early evening information meetings about the intervention program and the tests at Rigshospitalet. All the potential participants attending the information meetings were screened for inclusion.

Inclusion criteria was overweight defined as a BMI above the 90% percentile (thus also including obesity which is defined as a BMI above the 99% percentile) on the BMI curve (guidelines from the Danish Paediatric Society on age- and gender specific BMI curves) [20] at inclusion day. Subjects with an age below six and above 10 years, physical or mental handicap, use of medicine within the last three months, and somatic illness as cause for obesity were excluded. After inclusion the children were gender and age-stratified and block-randomized into an intervention or a control group by the Rockwool Foundation Research unit.

All baseline measurements were performed within the duration of four days, during the first week of January 2012. The subjects from the intervention group and the control group were equally divided among these four days. The follow up measurements were performed 20 weeks after baseline (in mid June 2012). Each child was tested during two days.

At both study days all the children obtained a medical examination, as well as blood screen, and pubertal developmental stage was evaluated by breast stages (B1-B5) in girls and by genital stages (G1-G5) in boys according to Tanners criteria. Furthermore, testicular volume was assessed by orchidometry, and a testicular volume above 3 mL was defined as the onset of puberty.

The recruitment began 5 December 2011, baseline measurements began 2 January 2012, the intervention began 9 January 2012, and the follow up measurements were completed 18 June 2012. All measurements were carried out at the Centre of Inflammation and Metabolism, Rigshospitalet, Denmark.

### Intervention

The intervention was conducted by a private association, and was initiated the week after baseline measurements. The intervention consisted of a) 60 minutes weekly group training session of the children at schools close to the children’s residences, b) 90 minutes weekly group training session of the children, their parents, and siblings at a municipal fitness club, c) individual nutritional guidance and coaching of the children and their families (twice during the program), and d) common cooking and dining with the children and their families (twice during the program). The weekly training session for the children alone began with a 15 minute talk about the past week and the well-being of the children, followed by 45 minutes of continual exercise, games, and dancing. The weekly training session for the families began with 30 minutes of instruction on health topics, e.g. a healthy diet and the consequences of overweight and physical inactivity. In the weekly sessions for the children alone, the training was focused on physical activity games, e.g. schoolyard games, whereas the weekly session with the families comprised primarily aerobic and strength exercises. Although the program was not based on any specific psychological strategies, the instructors continuously tried to give the children a positive and successful experience while progressively increasing the intensity of the physical activity and challenging the children’s motor skills. The nutritional guidance followed ‘The Official Dietary Guidelines’ by the Ministry of Food, Agriculture and Fisheries (Danish Veterinary and Food Administration) [21], and there were no caloric restrictions during the intervention. The program was free of charge for the families, as the program was fully paid for by funding, and the training facilities were made available by the Høje-Taastrup Municipality. The two instructors in the program were a bachelor in Nutrition and Health and an exercise instructor. After the follow up test days the control group entered the intervention program for 40 weeks.

### Outcome measures

#### Anthropometry and blood pressure measurements

Weight was measured at baseline and follow up to the nearest 0.1 kg on an electronic scale (Bisco scales, Farum, Denmark), with the children dressed in underwear. Height was measured to the nearest 0.5 cm using a portable stadiometer (HR 001, Tanita Leicester Portable Height Measure, Tokyo, Japan). Body mass index (BMI) was calculated as weight (kg)/height^2^ (m^2^) and standardized BMI (zBMI) was calculated for international comparison using the International Obesity Task Force (now also known as World Obesity/ Policy & Prevention) BMI cut-off values for thinness, overweight and obesity developed by Cole et al. [22]. Waist and hip circumference were measured by the same person at baseline and follow up to the nearest 0.5 cm with a circumference measuring tape (Meterex, Langenfeld, Germany). Waist circumference was defined as the midline between the lowest border of rib cage and the upper border of iliac crest, and hip circumference as the broadest level on hips while the child was standing. The waist-height ratio (WHtR) was calculated as waist (m)/height (m). The resting blood pressure and heart rate were measured three times with an automated oscillometric upper arm blood pressure monitor (Omron 705IT, Omron Corporation, Kyoto, Japan), and the mean of the last two measurements were used. Arterial hypertension was defined according to the guidelines from the Danish Paediatric Society [23].

#### Body composition measured by DXA scan

The same person performed the scans on all children at baseline and at follow up. Total and regional muscle and fat mass were measured using a dual-energy X-ray absorptiometry scanner (Lunar Prodigy Advance, GE Medical systems, Lunar, Milwaukee, WI, USA).

#### Physical activity measurements

The children’s habitual physical activity was assessed by a pedometer (Yamax Digi-walker CW-300 pedometer). The pedometer was worn attached to the waistband during all waking hours (except while bathing). A mean of the steps registered on the least active single day (minimum) and a mean of the steps registered on the most active single day (maximum) were calculated for each of the two groups in the two periods. To adjust for within-week variation in physical activity the total mean steps for each child were weighted with 5/7 and 2/7 for weekdays and weekend days, respectively.

Cardiorespiratory fitness was assessed indirectly using a modified Andersen fitness test [24]. In the modified Sargent Jump Test (using a vertical jump mat from SSN produced by Keld Jepsen, Denmark) the child was placed with two feet on the mat with a belt connected to a measuring tape grounded to the mat. The vertical jump was performed with free movements of the upper limbs and total freedom in joint flexion of the lower limbs. The child was asked to jump straight up as high as possible and land on both feet again, and the best result (in cm) of three attempts was recorded.

#### The standardized oral glucose tolerance test and the continuous glucose monitoring system

The children were asked to monitor their diet the day before the baseline study days, and subsequently eat exactly the same diet the day before the follow up study days. After a 10 hour overnight fast the children were transported to the laboratory. EMLA patches (AstraZeneca, London, England) were applied for approximately 30 minutes prior to peripheral catheter insertion, and the Continuous Glucose Monitoring System (CGMS) sensor placement. A small, peripheral catheter was placed in an antecubital vein for blood sampling. An oral glucose load was administered (1.75 g/kg, maximum of 75 g in 300 ml water flavored with lemon juice). Baseline screening samples were drawn at time point 0, and blood samples (glucose and insulin) were drawn every 30 minutes for two hours. Blood samples were collected in EDTA-containing tubes and immediately centrifuged for 15 minutes at 3,500 rpm, and the plasma was stored at -80°C until further analysis.

Pre-diabetes and diabetes cut-off values were defined by the International Society for Pediatric and Adolescent Diabetes [25]. HOMA-IR was calculated as fasting insulin concentration (μU/mL) X fasting glucose concentration (mmol/L)/ 22.5 [26, 27]. MATSUDA insulin sensitivity index was calculated as [28]:

The CGMS consisted of two systems: 1) The iPro2 recorder (Medtronic Diabetes Inc, Northridge, USA) and 2) The Guardian REAL-time CGM (Medtronic Diabetes Inc, Northridge, USA), using the same glucose sensor (Enlite Glucose Sensor, Medtronic Diabetes Inc, Northridge, USA). The glucose sensor was inserted just under the skin of the lower part of the abdomen. The child (or their parents) measured a calibrating blood sugar one hour after insertion, and subsequently four times a day in five days using a blood sugar apparatus (Contour, Bayer HealthCare). Data from two consecutive days were used for analyses.

#### Biochemical assays

The circulating levels of plasma insulin, plasma c-peptide, plasma glucose, plasma cholesterols, plasma triglycerides, plasma alanintransaminase (ALAT), plasma aspartattransaminase (ASAT), plasma thyroid hormones, hemoglobin in blood, and glycosylated hemoglobin (HbA1c) in blood were measured using standard techniques at the Department of Clinical Biochemistry, Rigshospitalet University Hospital, Copenhagen, Denmark. Plasma leptin and plasma adiponectin levels were measured using Meso Scale Discovery (MSD) electrochemiluminescence (Meso Scale Discovery, Gaithersburg, MD, USA). Serum resistin and serum visfatin levels were measured using high-sensitivity ELISA (human resistin # DRSN00, R & D Systems, Minneapolis, MN, USA, and human visfatin # K4907-100, BioVision, Mountain View, CA, USA). All samples were run in duplicates and the means were calculated. The intra-assay coefficients of variation (CV) were less than 20% for leptin, adiponectin, resistin, and visfatin according to the manufacturer. Levels of serum Follicle-stimulating hormone (FSH), Luteinizing hormone (LH), testosterone, estradiol, sex hormone-binding globulin (SHBG) [29, 30], IGF-I, and Insulin-like growth factor-binding protein 3 (IGFBP-3) [31, 32] were measured as previously described with validated methods using established pediatric reference ranges.

### Statistical analyses

Analysis on the primary outcome (BMI) was performed according to the intention-to-treat principle. All secondary analyses were performed as ‘complete-case-analyses’. As data was missing on two subjects, data was imputed (using the Stata command ‘mi impute’) on the primary outcome. Differences between the study groups at baseline were assessed by the student’s t-test or Wilcoxon’s rank-sum test, when appropriate. Intervention effects were analyzed using analyses of co-variance (ANCOVA). Analysis on the primary outcome (including the imputed data) was furthermore adjusted for missing data uncertainty (using the Stata command ‘mi estimate’). The intervention effect (abbreviated as DELTA in the text) was defined as the difference in changes between the group’s outcome variables adjusted for the respective baseline values. The analyses were adjusted for their respective baseline values in order to comply with potential regression towards the mean [33]. Standard regression diagnostics were performed, and significance was accepted as P <0.05 (two-tailed). Secondary outcomes are regarded as explorative and are thus not corrected for multiple testing. Data analyses were performed using Stata12 (StataCorp, TX, USA).

## Results

### Participants

**Figure 1:**
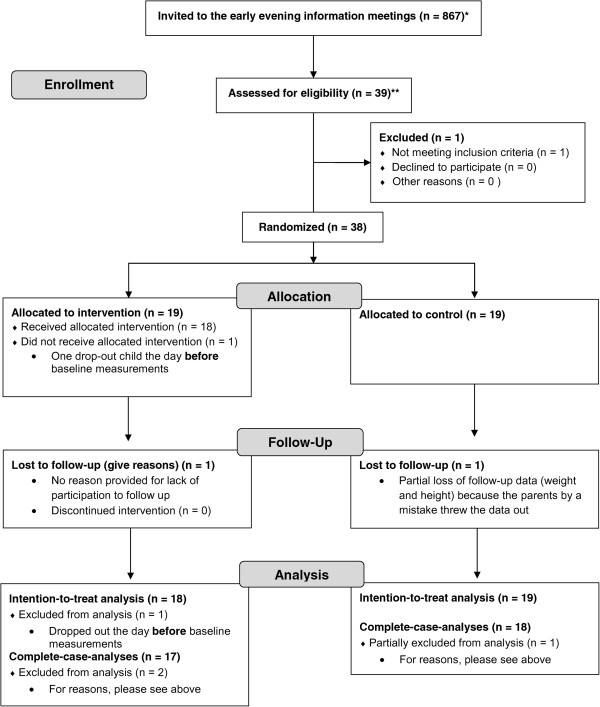
**Design of the randomized controlled trial.** CONSORT 2010 Flow Diagram [34] showing the number of children invited, enrolled, allocated, at Follow-Up, and in the analyses. *Children (age 7–10 years) at inclusion time in Høje Taastrup Municipality who were identified as overweight or obese by the ‘SundSkoleNettet’/‘The Healthy Schools Network’. **Children (age 7–10 years) at inclusion time who participated in the early evening information meetings.

Of the 39 children who came to the early evening information meetings, one child was excluded because of normal BMI, and 38 children fulfilled the inclusion criteria and were hence included in the study; 19 were allocated to the intervention group and 19 to the control group. The overall study design is depicted in Figure 1 (CONSORT 2010 Flow Diagram [34]).Before baseline measurements (see Figure 1) one child dropped out (n = 37 children at Baseline). Blood samples were only drawn in 30 of the 37 children at baseline, and due to logistical reasons the oral glucose tolerance test (OGTT) was only applied to a subsample of children (n = 26), and the CGMS on 20 children (one child was excluded because of lack of sufficient sensor data at baseline and three children excluded from analyses because of lack of sufficient sensor data at follow up). The anthropometric data from two children were lost, and the body weight is replaced with the weight from the DXA scans at baseline and follow up (one of these children was a drop-out at follow up). Six children were excluded from pedometer measurements because of illness or lack of data at baseline and further three in the analyses for the same reasons at follow up. Three children were excluded from analyses of the fitness test because of crying, walking or falling during the test, and one child from the jumping test because of lack of participation. None of these children deviated from the rest of the group in measurements of BMI and whole body dual-energy X-ray absorptiometry (DXA) scan.

The adherence (mean percentage of attended sessions) to the intervention program was 74 ± 17% (mean ± SD).

#### Baseline assessment

At baseline the 37 children had a mean age of 8.7 ± 0.9 years and a BMI of 21.8 ± 3.7 kg/m^2^. There were no significant differences between the control and intervention group on any of the descriptive baseline measurements (Table 1), and only in two of the explorative measurements (Table 2). The children had individually normal medical examinations and blood screens at baseline.

**Table 1:** **Descriptive characteristics of the children at baseline**

		Control group	Intervention group	P value
	n	Baseline	Baseline	
Girls *(total and percentage)*	37	*15 (83.3)*	*15 (78.9)*	-
Age (years)	37	8.7 (0.9)	8.8 (0.9)	0.8
Birth weight (gram)	35	3,634 (488.4)	3,555 (417.0)	0.6
**Body composition**				
Weight (kg)	37	39.7 (8.1)	44.6 (10.1)	0.1
BMI (kg/m2)	37	21.0 (3.5)	22.6 (3.8)	0.2
Overweight *(total and percentage)*	20	*12 (67)*	*8 (42)*	0.3
Obese *(total and percentage)*	17	*6 (33)*	*11 (58)*	0.1
zBMI	37	1.8 (0.8)	2.2 (0.8)	0.1
Waist (cm)	35	79.2 (7.4)	81.2 (8.9)	0.5
Hip (cm)	35	80.7 (8.7)	84.1 (8.2)	0.2
Waist-to-height ratio	35	0.6 (0.1)	0.6 (0.1)	0.9
**DXA scan**	**37**			
Lean body mass (kg)		23.1 (3.3)	25.3 (4.3)	0.1
Fat mass (kg)		15.0 (5.4)	17.6 (6.8)	0.2
Body fat tissue percent (%)		38.3 (7.1)	40.2 (7.0)	0.4
Android fat mass (kg)		1.3 (0.5)	1.7 (0.8)	0.08
Gynoid fat mass (kg)		2.9 (1.0)	3.4 (1.2)	0.2
Android/gynoid ratio		0.4 (0.1)	0.5 (0.1)	0.05
**Blood Pressure and pulse**	**37**			
Systolic (mmHg)		101.9 (8.0)	102.2 (8.9)	0.9
Diastolic (mmHg)		62.8 (7.7)	62.0 (6.1)	0.6
Heart Rate (beats/min)		81.4 (13.6)	79.7 (11.0)	0.7
**Fitness**				
Steps per day (maximum)	31	14,313 (3,176)	13,049 (2,783)	0.3
Modified Andersen fitness test (meter)	37	689.6 (135.5)	649.5 (173.9)	0.4
Jumping test (cm)	37	22.9 (4.3)	24.6 (6.3)	0.4

Presented as mean (standard deviation) or *total in the group (percentage of the group)*.

P value assessed by unpaired Student’s t-test.

zBMI, standardized BMI.

**Table 2:** **Descriptive characteristics of the explorative measurements at baseline**

		Control group	Intervention group	P value
	n	Baseline	Baseline	
**Glycemic control**				
HbA1c (mmol/mol)	30	34.9 (2.8)	34.9 (2.5)	1.0
**Oral glucose tolerance test**				
Fasting glucose (mmol/L)	30	4.3 (0.2)	4.4 (0.3)	0.2
Fasting insulin (mmol/L)	30	62.9 (38.5)	67.7 (25.4)	0.7
120 min (2 h) glucose (mmol/L)	26	5.7 (1.1)	5.6 (0.8)	0.8
Matsuda Index	25	21.8 (12.4)	13.0 (3.9)	P <0.05
HOMA-IR	30	1.7 (1.1)	1.9 (0.7)	0.6
**Mean 48 h BG (mmol/L) by CGMS**	19	4.8 (0.4)	5.3 (0.6)	P <0.05
**Biochemistry**				
Alanintransaminase (U/l)	29	30.1 (21.2)	26.8 (11.1)	0.6
Aspartattransaminase (U/l)	29	33.0 (12.2)	31.4 (5.2)	0.7
Thyrotropin (x10^-3^int.enh./l)	30	2.6 (1.2)	2.7 (0.9)	0.6
Triiodthyronin (nmol/l)	30	2.8 (0.5)	2.6 (0.3)	0.3
Thyroxin (nmol/l)	30	107.1 (26.8)	102.1 (18.9)	0.6
Total cholesterol (mmol/L)	30	4.6 (0.6)	4.3 (0.9)	0.5
LDL (mmol/L)	30	2.7 (0.4)	2.7 (0.8)	0.9
HDL (mmol/L)	30	1.5 (0.4)	1.4 (0.4)	0.5
Triglycerides (mmol/L)	30	0.9 (0.8)	0.7 (0.2)	0.4
Estradiol (pmol/L)^#^	29	35 (30)	28 (14)	0.05
Testosterone (nmol/L)^#^	28	0.12 (0.0)	0.12 (0.0)	0.9
Sex hormone-binding globulin (nmol/L)^#^	29	62 (37)	49 (33)	0.4
Follicle-stimulating hormone (IU/L)^#^	29	1.11 (1.33)	1.06 (0.81)	0.9
Luteinizing hormone (IU/L)^#^	29	0.03 (0.12)	0.03 (0.04)	0.4
IGFBP-3 (ng/mL)^#^	29	4080 (1130)	4695 (490)	0.06
Insulin-like growth factor 1 (ng/mL)^#^	29	229 (131)	244.5 (74)	0.6
Adiponectin (ng/mL)^#^	26	19,547 (8,790)	18,138 (5,740)	0.5
Leptin (pg/mL)^#^	25	17,613 (27,432)	26,399 (23,333)	0.3
Resistin (ng/mL)^#^	26	7.0 (2.5)	7.5 (2.1)	0.3
Visfatin (ng/mL)^#^	23	0.34 (0.49)	0.39 (0.62)	0.9

Presented as mean (standard deviation) and P value assessed by unpaired Student’s t-test, or #median (interquartile range) and P value assessed by Wilcoxon rank-sum test.

Mean 48 h BG by CGMS, mean blood glucose measured in 48 h by continuous glucose monitoring system.

HbA1c, glycosylated hemoglobin; *HDL*, high-density lipoprotein; *LDL*, low-density lipoprotein.

IGFBP-3, Insulin-like growth factor-binding protein 3.

#### Anthropometry and blood pressure measurements

The anthropometric data of the children at baseline is presented in Table 1. There was a decrease in total body weight and in BMI in the intervention group compared to the control group (Table 3). This included a significant increase of BMI points within the control group during the same period (Table 3). Waist, hip and WHtR measurements decreased in both groups, with a larger decrease in the intervention group compared to the control group (Table 3). One intervention child had arterial hypertension at baseline, and two control children and two intervention children were hypertensive at follow up. There were no significant changes in either blood pressure measurements or resting heart rate within or between the two groups from baseline to follow up (Table 3).

**Table 3:** **Delta changes in descriptive variables from baseline to follow up time point**

	n	Control ∆_adj_	n	Intervention ∆_adj_	n	Intervention effect ∆_adj_	P value
**Body composition**							
Weight (kg) *intention-to-treat*	18	1.9 (1.7; 2.1)	19	-1.9 (-2.1; -1.6)	37	-4.0 (-4.9; -3.0)	P <0.001
BMI (kg/m2) *intention-to-treat*	18	0.3 (0.3; 0.4)	19	-1.6 (-1.7; -1.5)	37	-2.0 (-2.5; -1.5)	P <0.001
zBMI *intention-to-treat*	18	0.07 (0.05; 0.08)	19	-0.43 (-0.44; -0.42)	37	-0.5 (-0.6; -0.4)	P <0.001
Weight (kg)	18	2.0 (1.4; 2.7)	18	-2.0 (-2.6; -1.3)	36	-4.0 (-4.9; -3.0)	P <0.001
BMI (kg/m2)	17	0.4 (0.0; 0.7)	18	-1.6 (-2.0; -1.3)	35	-2.0 (-2.5; -1.5)	P <0.001
zBMI	17	-0.07 (-0.2; 0.2)	18	-0.44 (-0.5; -0.3)	35	-0.5 (-0.6; -0.4)	P <0.001
Waist (cm)	17	-0.5 (-2.0; 0.9)	18	-5.3 (-6.7; -3.9)	35	-4.8 (-6.8; -2.8)	P <0.001
Hip (cm)	17	-0.7 (-2.1; 0.6)	18	-5.3 (-6.6; -4.0)	35	-4.5 (-6.4; -2.6)	P <0.001
Waist-to-height ratio	17	-0.01 (-0.02; 0.00)	18	-0.05 (-0.06; -0.04)	35	-0.03 (-0.05; -0.02)	P <0.001
**DXA scan**	**18**		**18**		**36**		
Lean body mass (g)		1,806 (1,352; 2,260)		1,043 (584; 1,501)		-763 (-1,426; -101,1)	P <0.05
Fat mass (g)		711 (185; 1,238)		-2,733 (-3,261; -2,205)		-3,344 (-4,200; -2,689)	P <0.001
Body fat tissue percent (%)		-0.4 (-1.5; 0.7)		-5.3 (-6.4; -4.2)		-4.9 (-6.5; -3.3)	P <0.001
Android fat mass (g)		53.8 (-33.8; 141.5)		-324.5 (-411.0; -237.9)		-378.3 (-503.6; -253.0)	P <0.001
Gynoid fat mass (g)		121.2 (5.0; 237.5)		-446.4 (-561.4; -331.5)		-567.7 (-732.8; -402.5)	P <0.001
Android/gynoid ratio		0.005 (-0.01; 0.02)		-0.044 (-0.06; -0.02)		-0.049 (-0.08; -0.02)	P <0.001
**Blood Pressure and pulse**	**18**		**18**		**36**		
Systolic (mmHg)		0.4 (-4.1; 5.0)		2.3 (-2.2; 6.9)		1.9 (-4.5; 8.4)	0.6
Diastolic (mmHg)		-0.2 (-5.1; 4.7)		-1.1 (-6.0; 3.7)		-0.9 (-7.8; 6.0)	0.8
Heart Rate (beats/min)		1.5 (-2.8; 5.8)		-4.2 (-8.5; 0.1)		-5.7 (-11.8; 0.4)	0.07
**Fitness**							
Steps per day (maximum)	14	2,760 (408; 5,111)	13	4,705 (2,314; 7,096)	27	1,946 (-1,140; 5,331)	0.3
Modified Andersen fitness test (meter)	17	62.3 (26.4; 98.3)	16	92.3 (55.3; 129.3)	33	30.0 (-22.0; 81.8)	0.3
Jumping test (cm)	18	1.9 (-0.3; 4.1)	17	4.0 (1.8; 6.2)	35	2.1 (-1.1; 5.2)	0.2

P values are representing the intervention effects (the difference in change between the groups) adjusted for the respective baseline values (95% confidence intervals).

P values assessed by ANCOVA.

zBMI, standardized BMI.

#### Body composition measured by DXA scan

While the intervention group significantly lost fat mass, the control group gained fat mass, from baseline to follow up (Table 3). Furthermore, there was a significant decrease in android and gynoid fat mass for the intervention group compared to the control group from baseline to follow up measurements (Table 3).

#### The glucose metabolism

**Figure 2:**
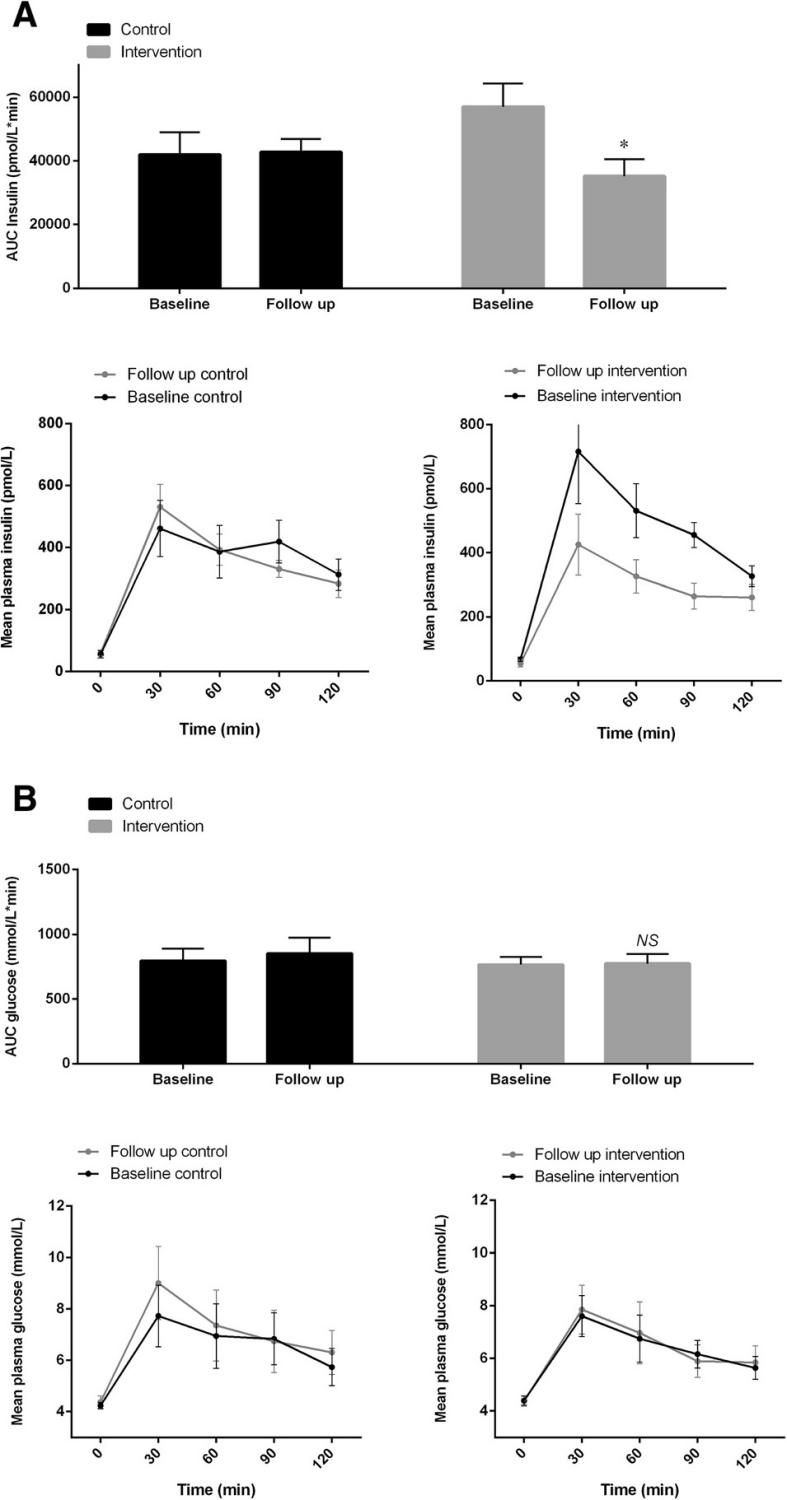
**The glucose metabolism measured by oral glucose tolerance test.** The area under curve is shown as bar graphs, while the response over time is shown as curves for plasma insulin **(A)** and glucose **(B)**, respectively. P values are representing the intervention effects (the difference in change between the groups) adjusted for the respective baseline values (95% confidence intervals as error bars on bar graphs and Standard Error of the Mean as error bars on curves). ★, *P* <0.05; *ns*, not significant.

None of the children were diabetic according to the standardized OGTT performed (Table 2). Three children were pre-diabetic (one control child at baseline, and two from the control group and one from the intervention group at follow up). There was a decrease in the area under curve (AUC) from baseline to follow up for plasma insulin, DELTA = -12,371 pmol/L*minutes (95% CI: -22,816; -1,926, P = 0.02, Figure 2A) and plasma C peptide, DELTA = -48,263 pmol/L*minutes (95% CI:-84,068; -12,459, P = 0.01, data not shown) during the OGTT in the intervention group compared to the control group with no difference in AUC plasma glucose (Figure 2B). Furthermore, there was an increase in the MATSUDA Index for insulin sensitivity at follow up in the intervention group compared to the control group (Table 4). There were no differences between the groups in regard to DELTA values of the fasting plasma levels of glucose, insulin, HbA1c or HOMA-IR (Table 4). Furthermore, there were no differences between the two groups in the glucose measurements during the CGMS (Table 4).

**Table 4:** **Delta changes in the explorative measurements from baseline to follow up time point**

	n	Control ∆_adj_	n	Intervention ∆_adj_	n	Intervention effect ∆_adj_	P value
**Glycemic control**							
HbA1c (mmol/mol)	12	-0.3 (-0.8; 0.1)	14	-0.5 (-0.9; -0.1)	26	-0.2 (-0.8; 0.5)	0.6
**Oral glucose tolerance test**							
Fasting glucose (mmol/L)	14	0.1 (-0.1;0.3)	14	0.1 (-0.1; 0.2)	28	0.0 (-0.3; 0.3)	0.4
Fasting insulin (mmol/L)	13	-4.7 (-17.6; 8.2)	12	-13.4 (-26.7; -0.1)	25	-8.7 (-27.4; 10.0)	0.3
120 min (2 h) glucose (mmol/L)	10	0.6 (-0.1; 1.3)	13	0.1 (-0.5; 0.7)	23	-0.5 (-1.4; 0.4)	0.3
Matsuda Index	9	-3.4 (-10.2; 3.5)	12	7.4 (1.9; 13.0)	21	10.8 (1.4; 20.2)	P <0.03
HOMA-IR	13	0.1 (-0.5; 0.3)	12	-0.4 (-0.8; 0.1)	25	-0.3 (-0.9; 0.3)	0.3
Mean 48 h BG (mmol/L) by CGMS	7	0.1 (-0.6; 0.9)	9	-0.3 (-1.0; 0.4)	16	-0.4 (-1.5; 0.7)	0.4
**Biochemistry**							
Alanintransaminase (U/l)	12	-7.9 (-12.6; -3.3)	14	-9.1 (-13.3; -4.8)	26	-1.1 (-7.4; 5.2)	0.7
Aspartattransaminase (U/l)	12	-2.0 (-5.1; 1.1)	14	-2.9 (-5.8; 0.0)	26	-0.9 (-5.1; 3.3)	0.7
Thyrotropin (x10^-3^int.enh./l)	14	0.1 (-0.4; 0.6)	14	0.3 (-0.1; 0.8)	28	0.2 (-0.5; 0.9)	0.5
Triiodthyronin (nmol/l)	14	-0.2 (-0.3; 0.0)	14	-0.2 (-0.4; -0.1)	28	-0.1 (-0.3; 0.1)	0.5
Thyroxin (nmol/l)	14	0.8 (-3.9; 5.5)	14	-0.6 (-5.3; 4.1)	28	-1.4 (-8.0; 5.3)	0.7
Total cholesterol (mmol/L)	14	-0.3 (-0.5; 0.0)	14	-0.5 (-0.8; -0.3)	28	-0.3 (-0.6; 0.0)	0.07
LDL (mmol/L)	14	-0.1 (-0.3; 0.1)	14	-0.4 (-0.6; -0.2)	28	-0.3 (-0.6; 0.0)	0.06
HDL (mmol/L)	14	0.1 (0.0; 0.1)	14	0.0 (-0.1; 0.1)	28	-0.1 (-0.2; 0.1)	0.4
Triglycerides (mmol/L)	14	-0.1 (-0.2; 0.0)	14	-0.3 (-0.4; -0.1)	28	-0.2 (-0.3; 0.0)	0.1
Estradiol (pmol/L)	13	-11.8 (-22.4; -1.2)	13	-14.8 (-25.5; -4.1)	26	-3.0 (-18.6; 12.5)	0.7
Testosterone (nmol/L)	12	0.0 (0.0; 0.1)	13	0.0 (0.0; 0.1)	25	0.00 (-0.1; 0.1)	1
Sex hormone-binding globulin (nmol/L)	13	4.2 (-2.4; 10.8)	13	17.0 (10.5; 23.5)	26	12.9 (3.5; 22.3)	P <0.01
Follicle-stimulating hormone (IU/L)	13	0.1 (-0.2; 0.3)	13	0.0 (-0.3; 0.2)	26	-0.1 (-0.4; 0.3)	0.7
Luteinizing hormone (IU/L)	13	0.0 (-0.1; 0.3)	13	0.0 (-0.2; 0.2)	26	-0.1 (-0.3; 0.2)	0.5
IGFBP-3 (ng/mL)	13	-73.2 (-233.5; 87.0)	13	-124.5 (-291.0; 42.0)	26	-51.3 (-293.0; 190.4)	0.7
Insulin-like growth factor 1 (ng/mL)	13	49.8 (15.1; 84.5)	13	4.4 (-30.2; 39.1)	26	-45.4 (-94.5; 3.8)	0.07
Adiponectin (ng/mL)	12	-1,693 (-3,143; -242)	13	595 (-801; 1,990)	25	2,287 (254; 4,320)	P <0.05
Leptin (pg/mL)	11	-7,803 (-15,402; -205)	14	-14,374 (-21,069; -7,679)	25	-6,570 (-16,944; 3,803)	0.2
Resistin (ng/mL)	12	-0.4 (-0.9; 0.1)	14	-0.6 (-1.0; -0.1)	26	-0.2 (-0.8; 0.5)	0.6
Visfatin (ng/mL)	10	0.0 (-0.3; 0.2)	11	0.2 (-0.1; 0.4)	21	0.2 (-0.2; 0.5)	0.3

P values are representing the intervention effects (the difference in change between the groups) adjusted for the respective baseline values (95% confidence intervals).

P values assessed by ANCOVA.

Mean 48 h BG (mmol/L) by CGMS, mean blood glucose measured in 48 h by continuous glucose monitoring system.

HbA1c, glycosylated hemoglobin; *HDL*, high-density lipoprotein; *LDL*, low-density lipoprotein; IGFBP-3, Insulin-like growth factor-binding protein 3.

#### Physical performance

**Figure 3:**
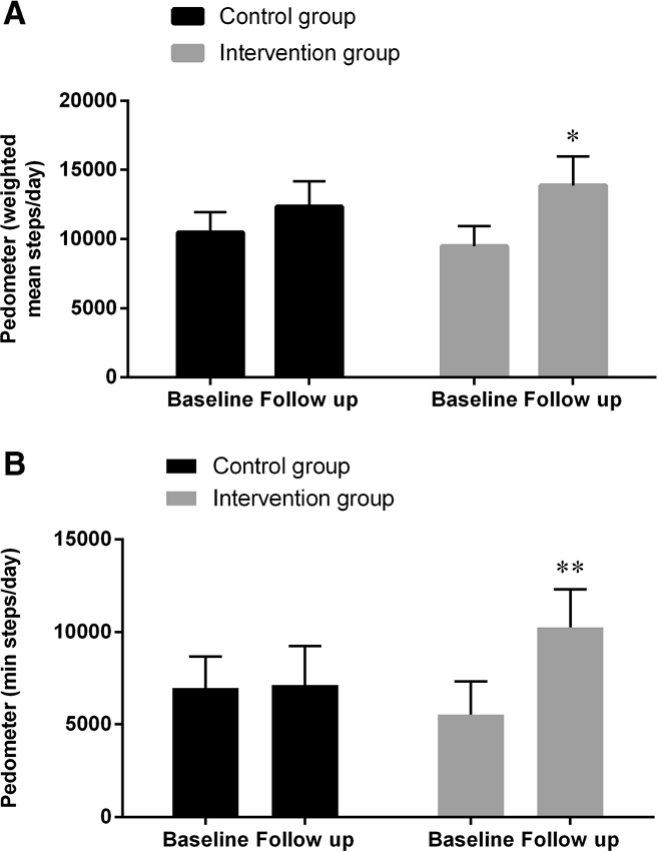
**Physical activity measured by pedometer.** Weighted mean steps per day **(A)** and mean minimum steps registered on the least active single day **(B)** registered by Pedometer. P values are representing the intervention effects (the difference in change between the groups) adjusted for the respective baseline values (95% Confidence intervals as error bars). ★, *P* <0.05; ★★, *P* <0.01.

There was an increase in weighted mean steps per day in both groups (P = 0.05 within the control group), but a significantly higher increase in the intervention group, with DELTA = 2,617 steps per day (95% CI: 202; 5,032, P = 0.04, Figure 3). In addition, there was an increase in the mean number of minimum steps per day in the intervention group, DELTA = 3,991 minimum steps/day (95% CI: 1,162; 6,820, P = 0.008, Figure 3) whereas there was no difference between groups with regard to mean maximum steps per day (Table 3). Both group enhanced performance from baseline to follow up with regard to the modified Andersen fitness test and the modified Sargent vertical jump test.

There were no significant differences at follow up in enhanced performance between the two groups in the two tests (Table 3).

#### Pubertal stages

The children in both groups were equally pre-pubertal/pubertal at baseline (intervention group = 3 and control group = 4) and there was no indication of the intervention delaying the pubertal development.

#### Biochemistry

There were no significant differences between the two groups at follow up with regard to hemoglobin (data not shown) in blood, plasma ALAT, plasma ASAT, plasma thyroid hormones, and plasma lipid levels (Table 4).

There was an increase in plasma levels of adiponectin in the intervention group compared to the control group at follow up (Table 4). A larger increase in SHBG was found in follow up measurements in the intervention group compared to the control group (Table 4). There were no significant DELTA differences between the two groups in plasma levels from baseline to follow up of serum resistin, plasma leptin, and serum visfatin (Table 4).

## Discussion

Findings from this trial indicate that a relatively low intensive multicomponent intervention can induce large and clinically important decreases in BMI and other measures of adiposity in pre-pubertal or early pubertal overweight children. In addition, this intervention induces changes in metabolic risk factors, involved in the pathogenesis of type 2 diabetes. Furthermore, the intervention seemed to increase the physical activity level among the intervention group by increasing their basal physical activity level (minimum number of steps/day). Thus, the present study adds important knowledge to the existing evidence of the effect from different interventions for overweight and obese children [17, 18, 35, 36].

Overweight and obesity are most commonly defined by age- and gender specific BMI percentiles in children [22]. Some studies argue that abdominal circumference is a better predictor for type 2 diabetes than BMI [12, 37]. Central (visceral) fat is greatly associated with cardiovascular diseases and type 2 diabetes [38]. Both physical inactivity and visceral fat are associated with chronic low-grade inflammation [2], and it has been shown that chronic inflammation contributes to the development of e.g. insulin resistance, atherosclerosis and tumor growth [2]. The amount of visceral fat is associated with obesity in children [39] as well as inactivity and high caloric intake in adults [9, 40]. WHtR in children and waist-to-hip-ratio in adults correlates with central (visceral) obesity [41] and has been shown to be more accurate than BMI in predicting cardiometabolic risk factors in both children and adults [42–44]. In the present study there was a significant decrease in WHtR, and thereby in central obesity, in the intervention group, indicating an important risk reduction with regard to cardiometabolic diseases within these children. The children in the present study had normal glucose metabolism at baseline and at follow up (normal fasting glucose, fasting insulin, and two hours glucose values during OGTT). There was however a significant decrease in insulin AUC during the OGTT and a significant increase in the MATSUDA insulin sensitivity index in the intervention group compared to the control group, indicating an improvement of the children’s peripheral insulin sensitivity, i.e. improvement of the peripheral utilization of glucose, which is consistent with the present finding of improved WHtR and android fat mass (i.e. less visceral fat). Interestingly, there was a significant increase in the SHBG levels of the intervention children at follow up, which also support the findings in this study of an improvement in insulin sensitivity and decrease in metabolic risk, as earlier shown in adults [45] and in children and adolescents [46, 47].

An increase in the intervention group was also observed with regard to the mean number of steps per day as well as in the mean number of minimum steps taken per day. These data indicate an increase in physical activity of the children, supporting the findings of improved peripheral insulin sensitivity and less visceral fat mass [9, 40].

All children (n = 36) improved cardiorespiratory fitness (within group; control group with 8.2% and intervention group with 18.5%) and leg strength (within group; control group with 9.4% and intervention group with 15.4%), and, supported by other studies, there was no significant improvement in the intervention group compared to the control group at follow up [35, 48]. Previous studies have found elevated blood pressure levels in approximately 35% of overweight and obese European children [49]. Only one of the 37 children (2.7%) in the present study had arterial hypertension at baseline, and there was no significant decrease in arterial blood pressure in the intervention group compared to the control group after the intervention. One explanation could be that in pre-pubertal children this diagnosis is missed in 55% of casual measurements compared to 24 hour blood pressure measurement [37]. Another explanation could be that the children in the present study were primarily overweight rather than obese.

The hypothesis of the present study was that the explorative measurements would support the beneficial effects of weight loss and increased physical activity as previously found in adults and children [36, 50–52]. The present study could not reproduce findings such as a reduction in blood lipids, blood pressure or glycosylated hemoglobin, as the reductions were found in both groups in these variables. A lack of change in the latter parameters may simply be ascribed to the fact that these metabolic and physiologic parameters were normal at baseline. Thus, although the children were overweight, their metabolism was not severely disturbed. Knowing that adipose tissue also serves as a large endocrine organ that secretes hormones, cytokines and proteins [53], and that weight loss is associated with a reduction in the secretion of inflammatory factors [51], the present study looked at different biomarkers that may predict diabetic risk in children [54]. Low plasma levels of the adipokine, adiponectin, have been shown to correlate with increased risk of insulin resistance in children [51, 54], and there was an increase in the intervention group compared to the control group (who had a significant decrease within the group). For both of the adipokines; serum resistin and plasma leptin (markers of insulin resistance [43, 54]), there was a significant decrease within the intervention group, but this was not significant compared to the control group, where there also was a (non-significant) decrease. Serum levels of the adipokine visfatin (which decreases with exercise training [52]) did not change within the two groups.

### Strengths and limitations

The strength of the study is the low intervention exposure (only twice-a-week training sessions), accessibility, and the low drop-out rate. Only two children (<6%) dropped out of the study of whom one child dropped out before the baseline measurements. This indicates that the approach described in this paper is highly feasible. Additional strengths include the high internal validity of the outcome variables.

A possible methodological weakness of the study could be a self-selection bias, as the families had to show up to the information meetings before selection to the study. This would limit the generalizability of our findings. Furthermore, as this was a convenience sample, no formal power calculation was performed to determine the sample size, thus increasing the risk of under powering our study. However, with the given sample size and variation in the primary outcome, we are able to detect a between group difference of 1.84 in BMI across the study period. Other possible limitations are that the follow up measurements were taken 20 weeks after the beginning of the 40-week intervention, and there is no information of the long-term outcome and possible health benefits of this intervention. It should be pointed out, though, that the intervention program is working with the local Municipalities and the school nurses, and, that this was the first time the intervention was systematically evaluated. The present results may help with the future accessibility of the intervention.

Combined, the explorative data showed positive effects in the intervention group, however, when the control group was taken into account, the observed intervention effects were not always statistically significant. Thus, for many parameters the control group demonstrated slight positive effects. Reasonable explanations for this trend could be that the explorative analyses were underpowered and due to common factors such as maturation and practice. In addition, the length of the intervention (20 weeks out of 40 weeks intervention) or seasonal variation (baseline was just after the mandatory Christmas school holiday) could explain this tendency. Furthermore, one could argue for a positive effect on the control children might be that they were selected for, and had chosen to attend, the intervention after the study measurements. Hence, the control children were motivated for a lifestyle change already at baseline. Most of the children in this study were girls (30 out of 37), which could bias the effect as to biological gender differences in fitness and fat mass and limit generalizability to the general overweight population. Gender effects were not found in adjusted analysis, but as the study wasn’t powered to stratify by gender this observation should be interpreted with caution (data not shown).

It would be highly relevant to repeat the intervention in a larger scale where focus is on generalizability, cost-effectiveness, and the long-term outcomes of the intervention.

## Conclusions

The present study demonstrates a feasible multi-component intervention with physical activity, diet counseling and family coaching of overweight and obese children in urban public schools at the age of 7–10 years. Twenty weeks of this low-cost, twice-a-week training session intervention, that included the families’ participation, reduced the children’s BMI, and more importantly, WHtR, fat mass, fat distribution, and increased their physical activity (by increasing their minimum steps per day). In addition, this study shows that physical activity and diet counseling have the same beneficial effects in children on the elements of the metabolic syndrome as shown in adults [55, 56], including an increase in the peripheral insulin sensitivity, bringing these children closer to adopting a healthier lifestyle.
